# Fibroblast Growth Factor Receptor 1 Activation in Mammary Tumor Cells Promotes Macrophage Recruitment in a CX3CL1-Dependent Manner

**DOI:** 10.1371/journal.pone.0045877

**Published:** 2012-09-24

**Authors:** Johanna R. Reed, Matthew D. Stone, Thomas C. Beadnell, Yungil Ryu, Timothy J. Griffin, Kathryn L. Schwertfeger

**Affiliations:** 1 Microbiology, Immunology, and Cancer Biology Graduate Program, University of Minnesota, Minneapolis, Minnesota, United States of America; 2 Biochemistry, Molecular Biology, and Biophysics, University of Minnesota, Minneapolis, Minnesota, United States of America; 3 Department of Laboratory Medicine and Pathology and Masonic Cancer Center, University of Minnesota, Minneapolis, Minnesota, United States of America; Baylor College of Medicine, United States of America

## Abstract

Tumor formation is an extensive process requiring complex interactions that involve both tumor cell-intrinsic pathways and soluble mediators within the microenvironment. Tumor cells exploit the intrinsic functions of many soluble molecules, including chemokines and their receptors, to regulate pro-tumorigenic phenotypes that are required for growth and progression of the primary tumor. Previous studies have shown that activation of inducible FGFR1 (iFGFR1) in mammary epithelial cells resulted in increased proliferation, migration, and invasion *in vitro* and tumor formation *in vivo*. These studies also demonstrated that iFGFR1 activation stimulated recruitment of macrophages to the epithelium where macrophages contributed to iFGFR1-mediated epithelial cell proliferation and angiogenesis. The studies presented here further utilize this model to identify the mechanisms that regulate FGFR1-induced macrophage recruitment. Results from this study elucidate a novel role for the inflammatory chemokine CX3CL1 in FGFR1-induced macrophage migration. Specifically, we illustrate that activation of both the inducible FGFR1 construct in mouse mammary epithelial cells and endogenous FGFR in the triple negative breast cancer cell line, HS578T, leads to expression of the chemokine CX3CL1. Furthermore, we demonstrate that FGFR-induced CX3CL1 is sufficient to recruit CX3CR1-expressing macrophages *in vitro*. Finally, blocking CX3CR1 *in vivo* leads to decreased iFGFR1-induced macrophage recruitment, which correlates with decreased angiogenesis. While CX3CL1 is a known target of FGF signaling in the wound healing environment, these studies demonstrate that FGFR activation also leads to induction of CX3CL1 in a tumor setting. Furthermore, these results define a novel role for CX3CL1 in promoting macrophage recruitment during mammary tumor formation, suggesting that the CX3CL1/CX3CR1 axis may represent a potential therapeutic approach for targeting breast cancers associated with high levels of tumor-associated macrophages.

## Introduction

Activation of oncogenes in tumor cells results in the release of soluble factors into the microenvironment [1], [2]. These factors then act on the tumor cells in an autocrine manner and on non-tumoral cells in a paracrine manner to promote tumor formation and progression [3], [4]. Using an inducible model of fibroblast growth factor receptor 1 (FGFR1), we have demonstrated that activation of FGFR1 in mammary epithelial cells induces a number of secreted factors that are capable of acting in both autocrine and paracrine manners to promote tumorigenesis [5], [6], [7]. FGFRs and their ligands, fibroblast growth factors (FGFs), have been linked to the development of human breast cancer [6], [8]. Specifically, FGFR1 is amplified in approximately 10% of breast cancer and is associated with early relapse and poor patient survival [6], [9], [10], [11]. Moreover, triple negative breast cancer cell lines are sensitive to FGFR inhibitors indicating that FGFR signaling promotes growth of triple negative breast cancer cells and may serve as a potential therapeutic target in this tumor subtype [12]. Because paracrine effects of FGF activity on stromal alterations during normal biological processes, such as angiogenesis and inflammation during wound healing, are well documented [13], [14], it is likely that FGFR activity within tumor cells leads to similar alterations within the tumor microenvironment. Understanding the paracrine effects of FGF signaling in a tumor setting is important for identifying effective therapeutic strategies to target cancers associated with high levels of FGF signaling.

Published studies have demonstrated that FGFR activation in various cell types, including epithelial cells and endothelial cells, leads to recruitment of leukocytes, with macrophages representing the most prevalent cell type [13], [15]. The role of macrophages in enhancing tumor formation and progression has been well studied [3], [16], [17], [18], [19]. After recruitment to tumors, macrophages promote tumor progression through a variety of mechanisms including induction of factors required for angiogenesis, such vascular endothelial growth factor (VEGF), and via production of growth factors and extracellular matrix remodeling proteins [19]. Macrophages have also been shown to enhance tumor cell invasion by paracrine loop mechanisms whereby macrophages secrete growth factors that bind to receptors located on nearby tumor cells causing upregulation of chemoattractants which then signal back to receptors expressed by macrophages [20]. Since macrophages are well known to contribute to tumor progression and metastasis once they reach the primary tumor site [3], [17], [19], [21], it is essential to identify the factors responsible for macrophage recruitment. Of significant interest is the involvement of chemokines and their receptors in cancer progression since the processes of tumor cell migration and metastasis share similarities to leukocyte trafficking, which is dependent on chemokine signaling [22]. Macrophages have previously been shown to express CX3CR1 both in mouse and human macrophage cell lines in culture as well as *in vivo* [23], [24], [25], [26]. Moreover, recent studies indicate a role for CX3CR1/CX3CL1 in mediating cross-talk between tumor cells and their surrounding microenvironment in models of lymphocytic leukemia, glioblastoma, neuroblastoma, pancreatic cancer, prostate cancer, and breast cancer [26], [27], [28], [29], [30]. CX3CL1 is a structurally unique chemokine and is currently the only known member of the CX3C family of chemokines [31], [32], [33]. Unlike other chemokines, CX3CL1 functions as a transmembrane protein that can be cleaved by metalloproteinases to a soluble protein [30], [31], [32], [34]. There are numerous implications for the membrane-anchored form of CX3CL1 in cell adhesion and leukocyte trafficking [35]. Both transmembrane and soluble forms of CX3CL1 bind to the only known G protein-coupled seven-transmembrane receptor for CX3CL1, CX3CR1 [30]. Since CX3CR1 is expressed on the surface of macrophages and cross-talk between it and CX3CL1 have known roles in a diversity of tumor types, the CX3CL1-CX3CR1 axis may represent a novel regulator of tumor-associated macrophage recruitment.

Our previous studies have demonstrated that activation of an inducible FGFR1 (iFGFR1) construct in mammary epithelial cells leads to macrophage recruitment both *in vitro* and *in vivo* [15]. The studies described here focus on identifying iFGFR1-induced secreted factors responsible for macrophage recruitment. We demonstrate that activation of iFGFR1 in mammary epithelial cells leads to increased production of soluble CX3CL1. Further studies validate CX3CL1 as a target of FGFR activity in the triple negative breast cancer cell line HS578T. Moreover, we demonstrate that FGFR-induced CX3CL1 promotes macrophage recruitment *in vitro* and in an established iFGFR1-driven mammary tumorigenesis model *in vivo*. The results from these studies demonstrate the ability of FGF signaling to induce chemokine production in a tumor setting. Furthermore, these results suggest a novel role for CX3CL1 in recruiting macrophages in early stages of mammary tumorigenesis.

## Results

### Production of Soluble Proteins Following iFGFR1 Activation Promotes Macrophage Recruitment *in vitro*

**Figure 1:**
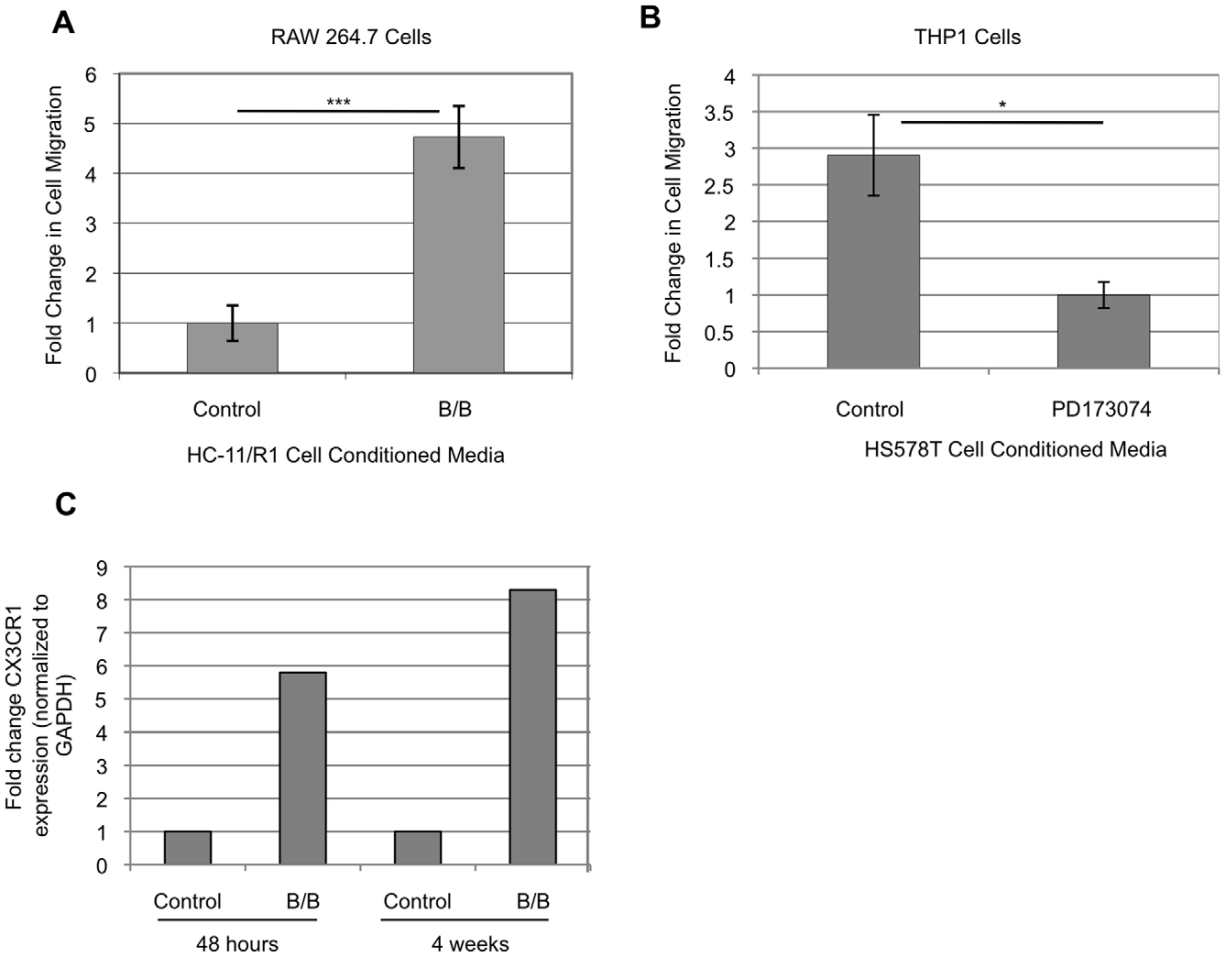
FGFR activation in mammary epithelial cells and breast cancer cells promotes macrophage recruitment. A) Conditioned media from HC-11/R1 cells containing soluble factors induced following treatment of cells with either B/B to activate iFGFR1 or ethanol as a solvent control for 24 were used to assess migration of RAW 264.7 cells. A significant induction in macrophage recruitment was observed in response to media containing iFGFR1-induced soluble factors. ***p<0.001. B) Conditioned media from HS578T cells treated with either PD173074, which inhibits FGFR activity, or DMSO as a solvent control were used to assess recruitment of THP-1 cells. A significant decrease in macrophage recruitment was observed in response to media from cells treated with an FGFR1 inhibitor. *p<0.05. Error bars represent SEM. C) Expression of CX3CR1 in macrophages isolated from mammary glands of mice treated with either B/B or solvent control for either 48 hours or 4 weeks. Expression levels were assessed using microarray analysis and expression levels are normalized to levels of GAPDH.

Progression of breast cancer is highly influenced by immune cells in the surrounding microenvironment, including macrophages [16], [36]. Therefore, it is important to determine the mediators responsible for recruiting macrophages to the site of the primary tumor during early stages of development. In previously published studies, we demonstrated that activation of an inducible FGFR1 that had been expressed in HC-11 mammary epithelial cells using retroviral transduction led to the production of soluble mediators that could promote macrophage recruitment *in vitro* [15]. The iFGFR1 construct is a variant of FGFR1 that lacks the extracellular ligand binding domain and is activated by binding of a synthetic dimerizer, B/B, which triggers homodimerization and ligand independent activation of downstream targets of FGFR1 signaling [37], [38]. More recent studies have utilized HC-11 cells that stably express the iFGFR1 construct (HC-11/R1) to further characterize the mechanisms by which FGFR1 activation promotes tumorigenesis [6], [15], [37]. To verify that the stably expressing HC-11/R1 cells behave in a similar manner to the retrovirally transduced cells used previously, the ability of iFGFR1 to produce soluble factors that promote macrophage recruitment was examined. As shown in Figure 1a, conditioned media obtained from HC-11/R1 cells treated with B/B to activate iFGFR1 for 24 hours led to increased recruitment of RAW 264.7 cells, a mouse macrophage cell line, in comparison with conditioned media from HC-11/R1 cells treated with a solvent control for 24 hours. Further studies were performed to validate the ability of endogenous FGFR signaling to induce soluble factors that promote macrophage recruitment using the FGF-responsive HS578T human breast cancer cell line [12]. Treatment of HS578T cells with the FGFR inhibitor PD173074 for 8 hours led to a decrease in the ability of conditioned media from these cells to promote recruitment of PMA-differentiated THP-1 macrophages, suggesting that FGFR activity regulates the production of soluble factors important for mediating macrophage recruitment by breast cancer cells (Figure 1b).

In order to determine potential mediators of macrophage recruitment, mass spectrometry was performed to identify soluble factors produced by HC-11/R1 cells. For these studies, HC-11/R1 cells were serum starved and then treated with ethanol as the solvent control or B/B to activate iFGFR1 for 24 hours. After treatment, conditioned media were collected and analyzed by mass spectrometry for the presence of soluble proteins produced by B/B-treated HC-11/R1 cells. Interestingly, mass spectrometric analysis revealed the presence of the chemokine CX3CL1 in the collected conditioned media. Several peptides representing this protein were identified at high confidence. CX3CL1 was identified in all three biological replicates tested with each unique peptide found at least twice (Table 1). CX3CL1 was also detectable in the solvent control samples (data not shown). However, because of the relatively low amount of overall spectra identified specific to this protein in both experiments, it was difficult to confidently determine a relative expression difference. Therefore, further studies were performed to determine whether CX3CL1 represents a viable candidate for macrophage recruitment. Microarray analysis performed on macrophages isolated from the mammary glands of MMTV-iFGFR1 transgenic mice treated with B/B demonstrated increased expression of CX3CR1, the sole receptor for CX3CL1, in macrophages compared with macrophages isolated from mammary glands of mice treated with solvent alone (Figure 1c), suggesting an increase in CX3CR1-positive macrophages in the mammary gland following iFGFR1 activation. Because CX3CL1 is a known chemoattractant for monocytes and macrophages [39], [40], these results led us to further examine the possibility that the CX3CL1/CX3CR1 axis is involved in FGFR1-induced macrophage recruitment.

**Table 1:** Proteomic analysis of soluble proteins after iFGFR1 activation.

Amino Acid Sequence	Peptide Probability	Precursor Mass Tolerance (ppm)	Number of Matched b and yIons/Total b and yIons Possible	Number of Times Identified from Three Experiments
(R)AIVLETTQHR(R)	95%	1.16	14/18	3
(K)HLDHQAAALTK(N)	95%	1.70	14/20	3
(R)IPVALLIR(Y)	95%	0.95	13/14	3
(R)YQLNQESC*GK(R)	95%	−1.88	16/18	2
(R)YQLNQESC*GKR(A)	95%	−0.80	17/20	2
(K)SLGSEEINPVHTDNFQER(G)	95%	−1.60	16/34	2

a The statistics listed in the table for each identified sequence represent those from the best matching spectrum.

* Carbamidomethyl modified cysteine.

HC-11/R1 cells were treated with B/B for 24 hours. Following treatment, conditioned media were collected and analyzed by mass spectrometry for soluble protein expression. Proteomic analysis indicated that several peptides representing CX3CL1 were present in the media following iFGFR1 activation.

### Activation of iFGFR1 in HC-11/R1 Mammary Epithelial Cells Induces Production of CX3CL1 via the NFκB Pathway

**Figure 2:**
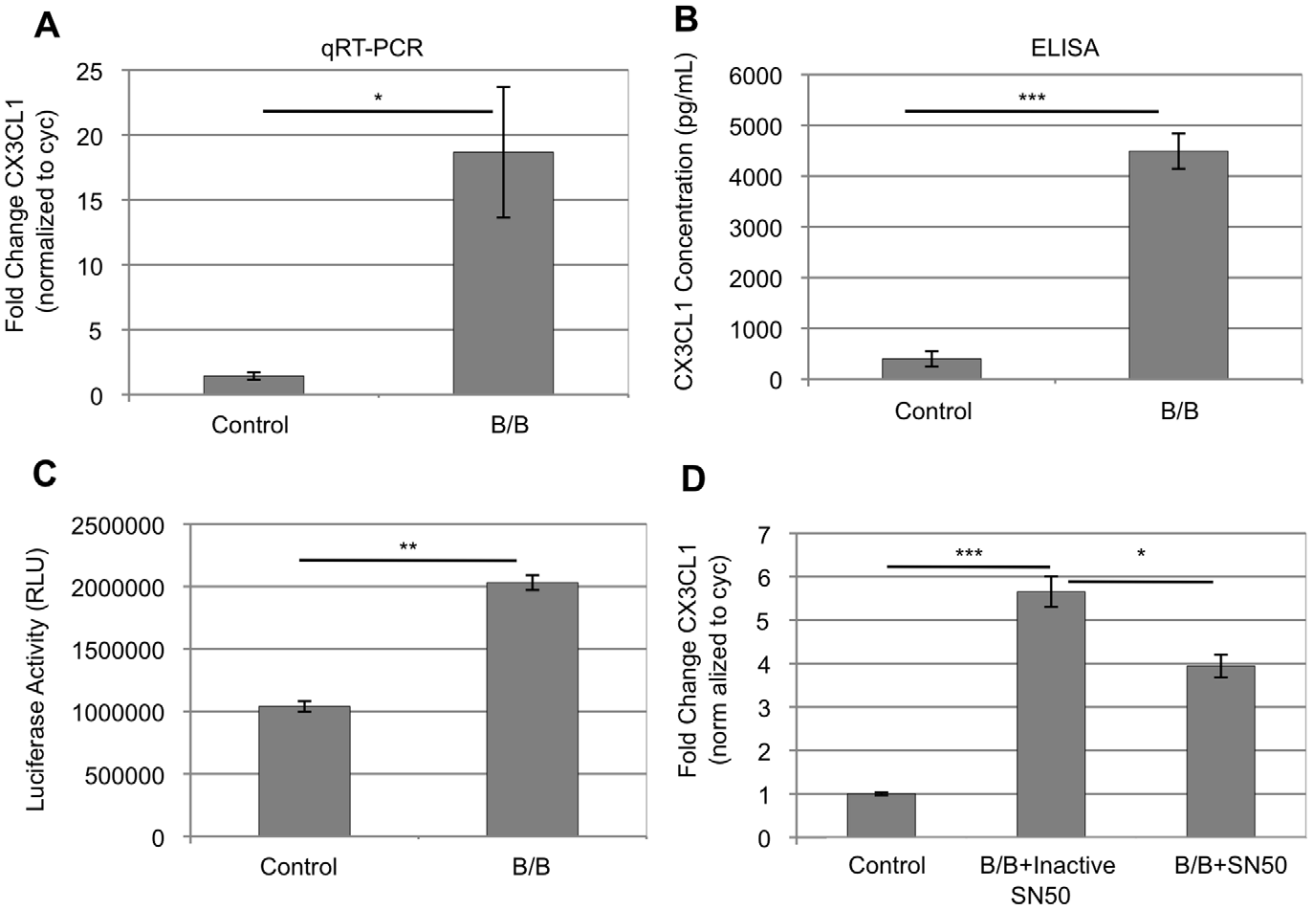
iFGFR1 activation induces secretion of CX3CL1 via the NFκB pathway. Treatment of HC-11/R1 cells with B/B induces production and secretion of CX3CL1. A) CX3CL1 gene expression was induced after 4 hours of B/B treatment as determined by qRT-PCR analysis. CX3CL1 levels were normalized to cyclophilin. B) ELISA analysis demonstrated that CX3CL1 is secreted by HC-11/R1 cells after 24 hours of B/B treatment. C) Treatment of HC-11/R1 cells with B/B for 6 hours increased the transcriptional activity of NFκB as measured by luciferase assay. D) Treatment of HC-11/R1 cells with the NFκB inhibitor peptide SN50 in conjunction with B/B significantly reduced CX3CL1 transcript levels as measured by qRT-PCR in comparison to HC-11/R1 cells treated with inactive SN50 in the presence of B/B demonstrating that iFGFR1-induced CX3CL1 is mediated by NFκB signaling. *p<0.05, **p<0.001, ***p<0.0001. Results in each figure panel are representative of a minimum of three different experiments. Error bars represent SEM.

To determine whether activation of iFGFR1 leads to increased levels of soluble CX3CL1, we treated HC-11/R1 cells with B/B to activate iFGFR1 and examined the expression levels of CX3CL1 in the conditioned media. Treatment with B/B significantly induced gene expression of CX3CL1 after 4 hours of treatment as measured by quantitative RT-PCR (qRT-PCR) (Figure 2a). Moreover, soluble protein levels of CX3CL1 were significantly elevated after 24 hours of B/B treatment (Figure 2b). Published studies have suggested that CX3CL1 expression is mediated by NFκB signaling [41], [42], [43]. Therefore, we examined the ability of iFGFR1 activation to signal through the NFκB pathway to promote gene regulation of CX3CL1. Initial studies were performed to determine the ability of iFGFR1 to activate NFκB using a NFκB luciferase-based reporter assay. Treatment of HC-11/R1 cells with B/B led to increased transcriptional activity of NFκB after 6 hours as indicated by significantly enhanced luciferase reporter gene expression (Figure 2c). These results were confirmed using an NFκB ELISA-based assay where treatment of HC-11/R1 cells with B/B for 6 hours significantly increased NFκB transcriptional activity (data not shown). Moreover, blocking signaling through the NFκB pathway with the NFκB-specific inhibitor peptide SN50 [44] resulted in partially but significantly reduced levels of CX3CL1 transcript after 4 hours of treatment with B/B in the presence of SN50 when compared to inactive control peptide in the presence B/B treatment (Figure 2d). Together, these studies demonstrate that CX3CL1 is a novel gene target of the iFGFR1/NFκB pathway in mammary epithelial cells.

### iFGFR1 Activation in Mouse Mammary Epithelial Cells Promotes Macrophage Migration via Secretion of CX3CL1

**Figure 3:**
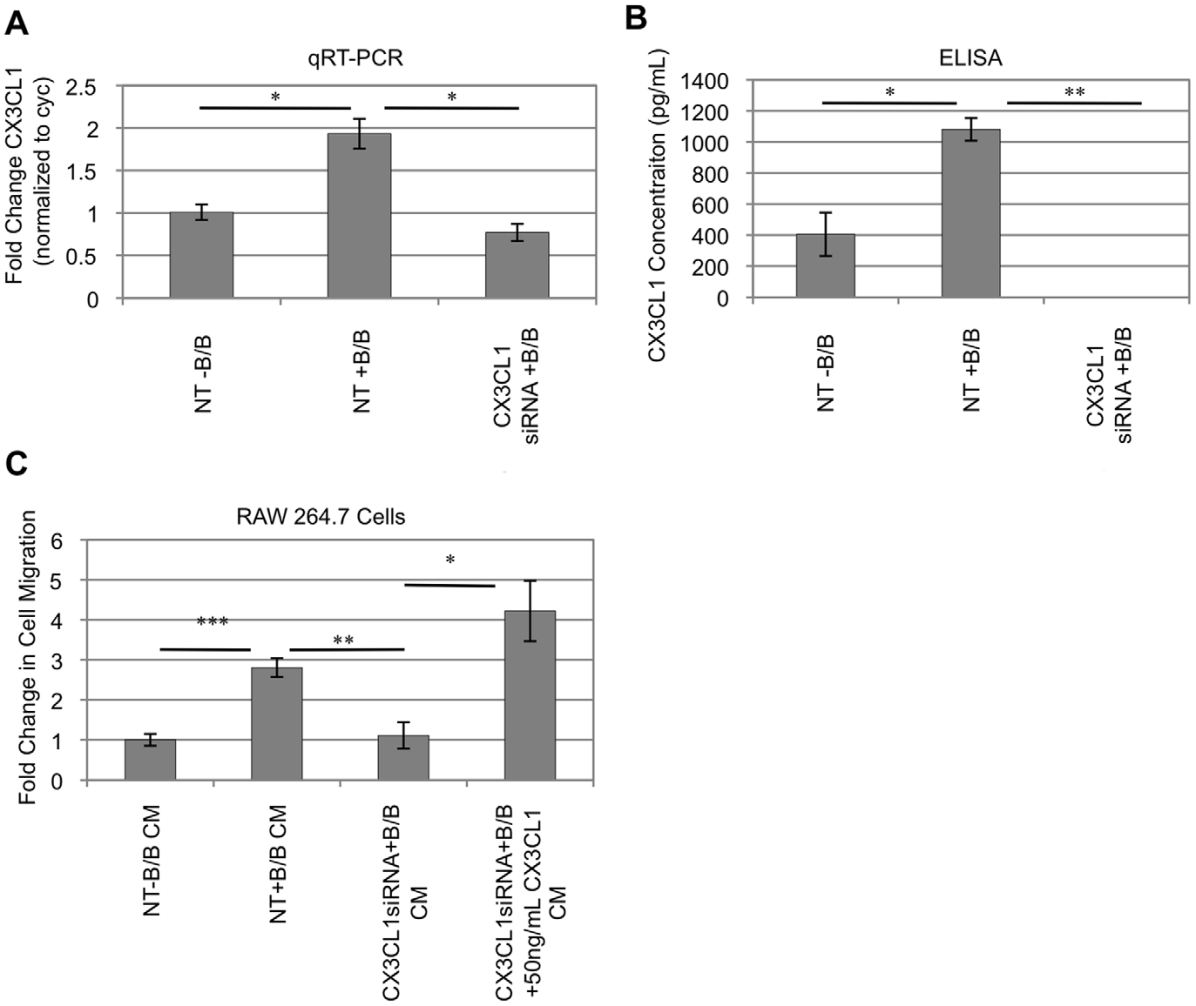
iFGFR1-induced CX3CL1 secretion by epithelial cells mediates macrophage migration. siRNA techniques were used to directly target CX3CL1 in order to determine the role of CX3CL1 in iFGFR1-mediated macrophage recruitment. A) CX3CL1 gene expression levels were significantly elevated in HC-11/R1 cells transfected with non-targeting (NT) siRNA. In comparison, CX3CL1 gene expression levels were significantly reduced in CX3CL1siRNA HC-11/R1 cells treated with B/B after 24 hours. B) ELISA was performed to verify reduction in CX3CL1 protein secretion in HC-11/R1 cells transfected with CX3CL1siRNA. Results indicated that soluble CX3CL1 protein concentrations were significantly induced in HC-11/R1 cells transfected with non-targeting (NT) siRNA. In comparison, soluble protein concentrations were reduced in CX3CL1siRNA HC-11/R1 cells treated with B/B after 24 hours. C) Conditioned medium from non-targeting HC-11/R1 cells treated with B/B significantly increased migration of RAW 264.7 cells relative to conditioned medium from cells treated with solvent alone. Exposure to conditioned medium from CX3CL1 siRNA HC-11/R1 cells significantly decreased RAW 264.7 cell migration. Furthermore, addition of 50ng/mL rmCX3CL1 to CX3CL1siRNA HC-11/R1 cells at the time of B/B treatment rescued the loss of RAW 264.7 cell migration in cells exposed to conditioned medium from CX3CL1siRNA HC-11/R1 cells. These results indicate that CX3CL1 is a key mediator of macrophage recruitment. *p<0.05, **p<0.001, ***p<0.0001. Results in each figure panel are representative of a minimum of three different experiments. Error bars represent SEM.

Based on the ability of CX3CL1 to recruit monocytes/macrophages [39], [40], we hypothesized that soluble CX3CL1 promotes iFGFR1-induced macrophage recruitment. Therefore, siRNA techniques were used to block the production of CX3CL1 in HC-11/R1 cells. Analysis of CX3CL1 gene expression levels by qRT-PCR indicate that CX3CL1 was transcribed upon activation of iFGFR1 by 24 hours of B/B treatment in the presence of a non-targeting siRNA, and that this induction of expression was significantly inhibited by a CX3CL1-specific siRNA (Figure 3a). Furthermore, soluble CX3CL1 protein levels were significantly reduced in B/B-treated CX3CL1 siRNA HC-11/R1 cells when compared to the levels produced by HC-11/R1 non-targeting control cells (Figure 3b). Conditioned media from CX3CL1 siRNA HC-11/R1 cells and non-targeting control cells were then used to measure the requirement for CX3CL1 in promoting migration of RAW 264.7 cells, which have been shown to express the sole receptor for CX3CL1, CX3CR1 [25]. Migration of RAW 264.7 cells was significantly increased in the presence of conditioned medium from B/B-treated HC-11/R1 cells expressing the non-targeting control siRNA in comparison to solvent treated non-targeting HC-11/R1 cells (Figure 3c). Furthermore, RAW 264.7 cell migration was significantly reduced in the presence of conditioned medium from B/B-treated CX3CL1 siRNA HC-11/R1 cells (Figure 3c). To further confirm the direct effect of CX3CL1 on RAW 264.7 cell migration, 50 ng/mL recombinant CX3CL1 protein was added to HC-11/R1 cells transfected with CX3CL1 siRNA. Addition of recombinant CX3CL1 significantly increased RAW 264.7 cell migration in comparison to CX3CL1 siRNA HC-11/R1 cells (Figure 3c). These results demonstrate that production of soluble CX3CL1 is critical for iFGFR1-mediated macrophage recruitment *in vitro*.

### Human HS578T Breast Cancer Cells Secrete CX3CL1 in an FGF-Dependent Manner to Promote Macrophage Cell Migration

**Figure 4:**
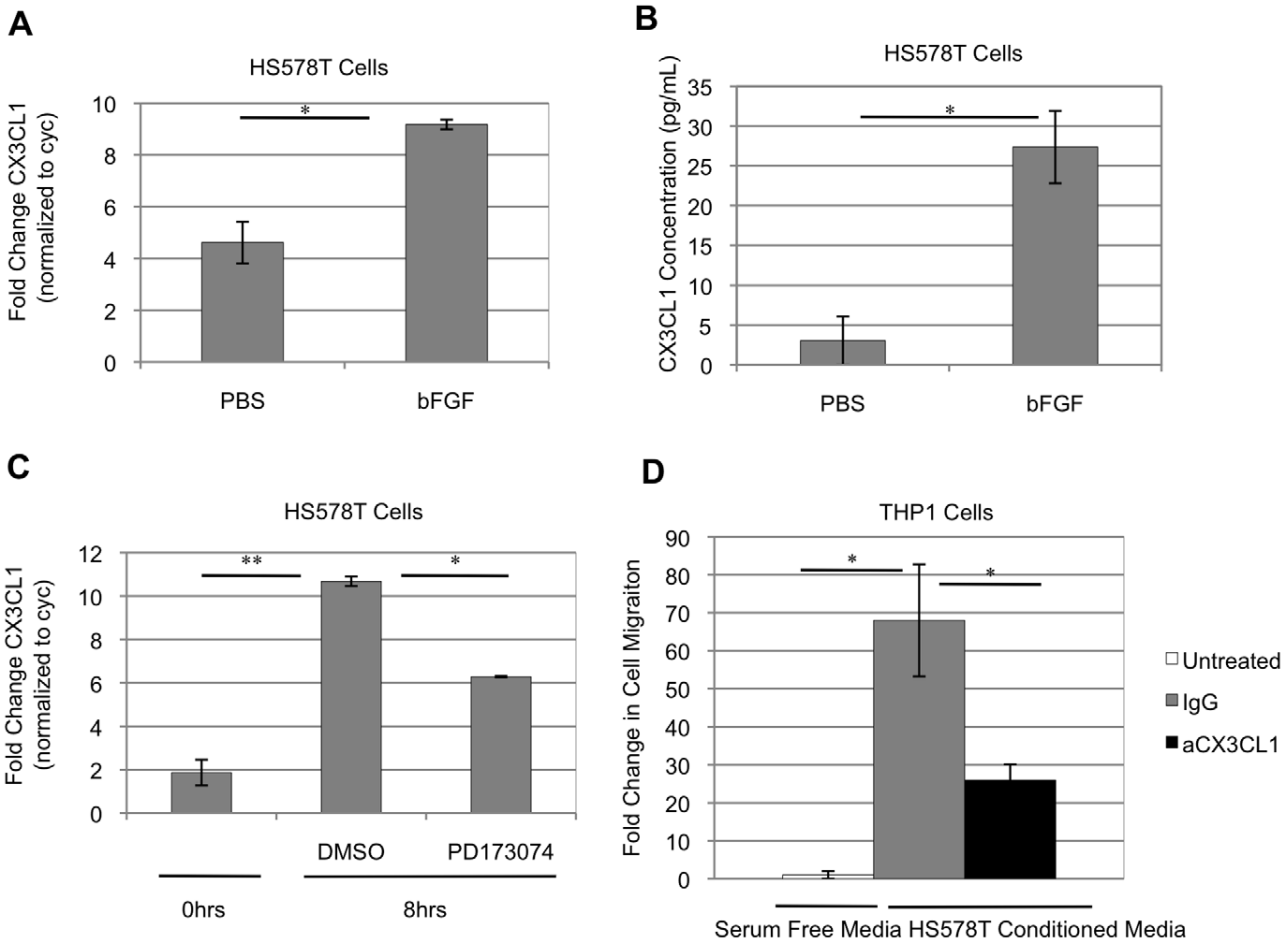
Human breast cancer cells secrete CX3CL1 in an FGF-dependent manner to promote macrophage cell migration. FGF-dependent production and secretion of CX3CL1 by HS578T cells mediates recruitment of human macrophages. A) HS578T cells treated with 50ng/mL bFGF demonstrated significant induction of CX3CL1 gene expression after 4 hours relative to PBS solvent control-treated cells. B) Soluble CX3CL1 protein concentrations were significantly upregulated in HS578T cells treated with bFGF for 8 hours in comparison to control-treated cells. C) HS578T cells, which produce high levels of endogenous bFGF, were treated with the PD173074 for 8 hours to inhibit autocrine FGFR activation. A significant reduction in CX3CL1 gene expression was observed relative to DMSO-treated cells as measured by qRT-PCR. D) THP1 cells that had been differentiated into macrophages using PMA demonstrated increased migratory potential when exposed to conditioned medium from CX3CL1-expressing HS578T cells in comparison to serum free medium. Furthermore, this enhanced cell migration was significantly reduced when THP1 cells were treated with a CX3CL1 blocking antibody (aCX3CL1) relative to IgG-treated THP1 cells. *p<0.05, **p<0.001. Results in each figure panel are representative of a minimum of three different experiments. Error bars represent SEM.

The human breast cancer cell line HS578T is dependent on FGFR signaling for proliferation and survival [12]. Based on the link between iFGFR1 activation and CX3CL1 production, further studies were pursued to determine the ability of endogenous FGFR activation to stimulate the production of soluble CX3CL1. For these studies, HS578T cells were serum starved for approximately 18 hours and then treated with basic FGF (bFGF). After 4 hours of bFGF treatment, HS578T cells demonstrated a significant elevation in CX3CL1 gene expression as determined by qRT-PCR analysis (Figure 4a). Furthermore, after 8 hours of treatment with bFGF, HS578T cells demonstrated an increase in production of soluble CX3CL1 protein to levels significantly higher than HS578T cells treated with PBS solvent control (Figure 4b). HS578T cells are known to produce endogenous bFGF, resulting in activation of FGFR signaling in an autocrine manner [12]. To further confirm that HS578T cells express CX3CL1 in an FGFR-dependent manner, HS578T cells were cultured in normal growth medium and were then treated with the FGFR inhibitor PD173074 to inhibit autocrine activation of FGFR. After 8 hours of treatment with PD173074, HS578T cells demonstrated a partial but significant decrease in CX3CL1 gene expression relative to the DMSO-treated control cells (Figure 4c). Moreover, the level of CX3CL1 gene expression was significantly elevated in DMSO-treated control cells relative to the baseline level of CX3CL1 gene expression measured at the time of PD173074 treatment (Figure 4c).

To determine whether CX3CL1 promotes HS578T cell mediated macrophage recruitment, PMA-differentiated THP1 cells were exposed to conditioned medium from HS578T human breast cancer cells, or serum free medium as a control, in a transwell assay. THP1 cells treated with goat IgG isotype control demonstrated a significant increase in migration in response to HS578T cell conditioned medium relative to cells exposed to serum free medium (Figure 4d). Treating THP1 cells with purified goat anti-CX3CL1 antibody in the presence of HS578T cell conditioned medium significantly reduced THP1 cell migration (Figure 4d). These results demonstrate that CX3CL1 secreted by HS578T cells in an FGF-dependent manner increases the migratory potential of macrophages and validates the link between iFGFR1 and CX3CL1 observed in the mouse cells.

### iFGFR1 Activation Promotes Recruitment of Macrophages in a CX3CR1-dependent Manner *in vivo*

**Figure 5:**
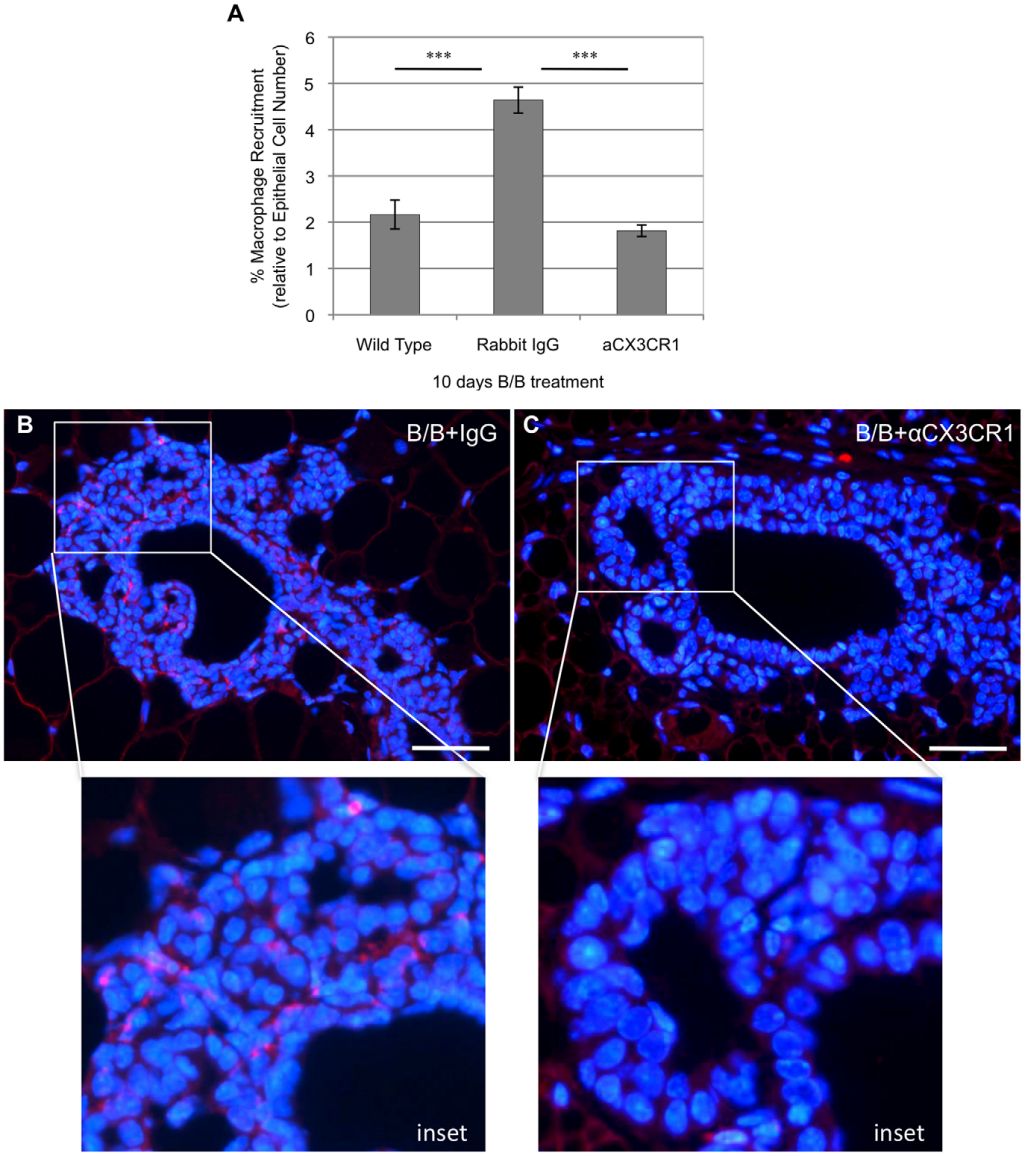
iFGFR1 activation *in vivo* promotes recruitment of CX3CR1-positive macrophages. MMTV-iFGFR1 transgenic mice were treated with B/B in order to analyze the population of macrophages that are recruited to the mammary epithelium during early stages of iFGFR1-induced mammary tumorigenesis. A) MMTV-iFGFR1 mice treated with B/B demonstrated an increase in macrophage recruitment after 10 days as indicated by an increased in the number of F4/80 positive cells. MMTV-iFGFR1 mice treated with anti-CX3CR1 in conjunction with B/B demonstrated a reduction in macrophage recruitment at 10 days indicating that iFGFR1 activation is responsible for recruiting a subset of macrophages that express CX3CR1. ***p<0.0001. Error bars represent SEM. B) Representative image of macrophages associated with budding epithelial structures in mammary glands from mice treated with control IgG antibody. C) Representative image of macrophages associated with budding epithelial structures in mammary glands from mice treated with anti-CX3CR1. Red = F4/80 staining, blue = DAPI. Scale bars represent 50 µM. Results in each figure panel are representative of a minimum of three different mice for each treatment group and genotype.

It has previously been shown that there is a significant influx of macrophages that are recruited to the mammary epithelium shortly after iFGFR1 activation [15]. To determine whether the CX3CL1/CX3CR1 axis is involved in iFGFR1-induced macrophage recruitment, transgenic MMTV-iFGFR1 mice were treated with a purified CX3CR1 neutralizing antibody. After 24 hours of treatment with anti-CX3CR1, mice were given B/B in conjunction with the CX3CR1 antibody, or IgG control, for 10 days. Results from immunofluorescent analysis using the macrophage-specific antibody F4/80 indicated that there was a significant influx of macrophages recruited to the mammary gland following 10 days of iFGFR1 activation (Figure 5a,b). Moreover, treatment with the CX3CR1 blocking antibody significantly decreased the number of macrophages that were recruited to the mammary gland (Figure 5a,c). These results suggest that iFGFR1 activation in mammary epithelial cells promotes recruitment of macrophages in a CX3CR1-dependent manner *in vivo*.

### Decreased Macrophage Recruitment Correlates with Decreased Angiogenesis

**Figure 6:**
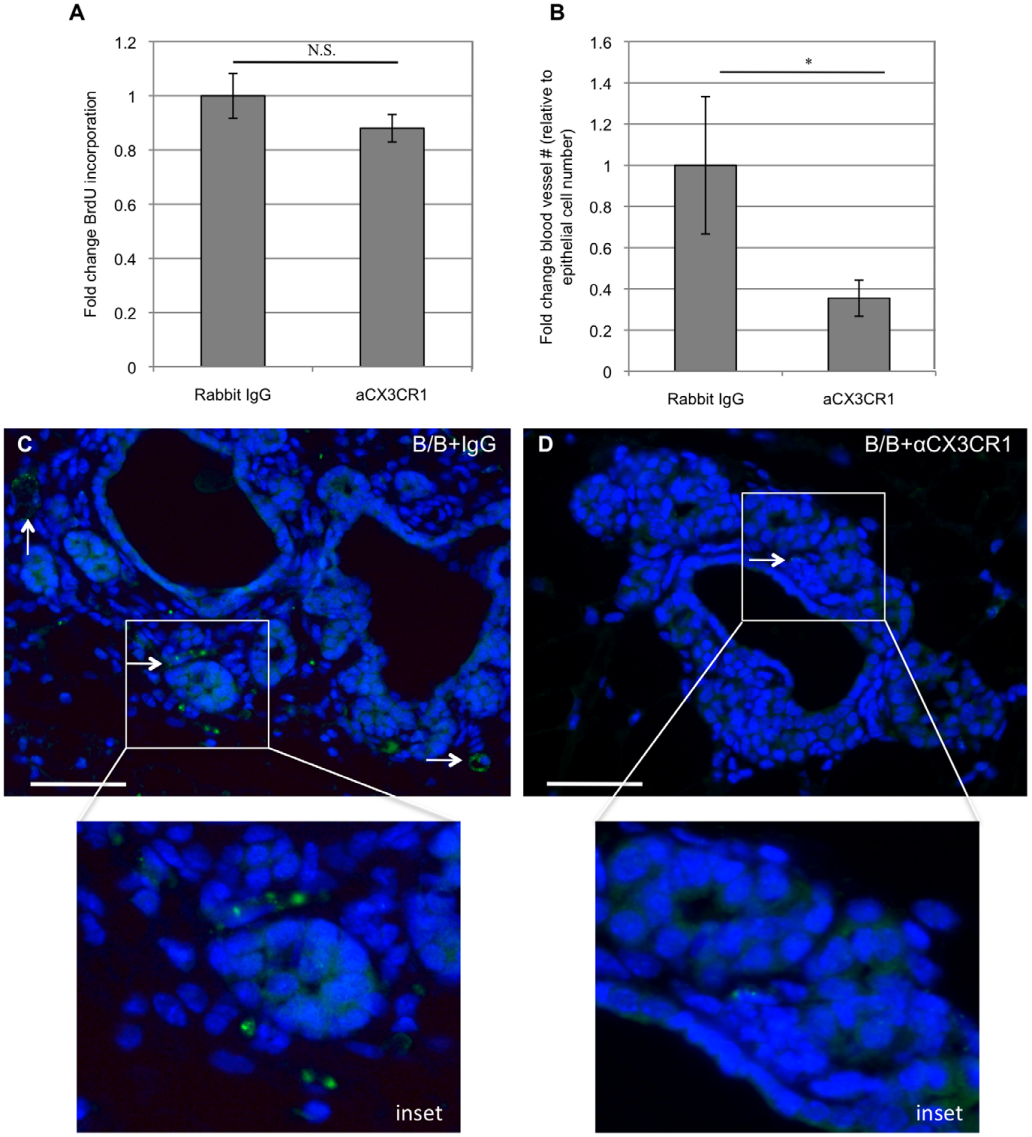
Decreased macrophage recruitment correlates with decreased angiogenesis. A) Quantification of BrdU incorporation demonstrates that decreased macrophage infiltration does not significantly correlate with a change in epithelial cell proliferation (N.S. = not significant). B) Quantification of VWF staining reveals decreased blood vessels associated with epithelial structures in mammary glands from mice treated with B/B and anti-CX3CR1 blocking antibody compared with mice treated with B/B and IgG control antibody. *p<0.05. C, D) Representative images of VWF stained mammary glands from mice treated with B/B and either control IgG antibody (C) or anti-CX3CR1 (D). Green = VWF staining, blue = DAPI. Arrows indicate VWF-positive structures. Scale bars represent 50 µM. Results in each figure panel are representative of a minimum of three different mice for each treatment group and genotype.

In previous studies, we demonstrated that macrophage depletion led to decreased iFGFR1-induced epithelial cell proliferation and angiogenesis [15]. To determine whether decrease in macrophage infiltration by blocking CX3CR1 correlates with these phenotypes, proliferation was assessed by analyzing BrdU incorporation and angiogenesis was assessed by immunostaining with an antibody to von Willebrand factor (vWF). Interestingly, there was not a significant difference in iFGFR1-induced epithelial cell proliferation in the mammary glands from mice treated with the CX3CR1 blocking antibody (Figure 6a). However, there was a decrease in the number of small blood vessels associated with epithelial structures in mammary glands from mice treated with the blocking antibody (Figure 6, b–d). These results suggest that blocking CX3CR1 leads to decreased macrophage infiltration, which correlates with a decrease in angiogenesis but not epithelial cell proliferation.

## Discussion

We have previously demonstrated that the inducible FGFR1 model of early stage mammary tumorigenesis can be utilized to understand the mechanisms involved in regulating various tumorigenic phenotypes, including proliferation, migration, and invasion [6], [37]. In this study, we used this model to define the mechanisms that regulate migration of cells in the surrounding tumor microenvironment following activation of an oncogenic stimulus. Specifically, we show that activation of the tyrosine kinase receptor FGFR1 induced CX3CL1 production and secretion in genetically altered mammary epithelial cells via NFκB signaling. Furthermore, epithelial cell-secreted CX3CL1 enhanced macrophage recruitment to the mammary epithelium during early stages of mammary tumorigenesis both *in vivo* and *in vitro*.

In order to better understand the novel role of iFGFR1-mediated CX3CL1 in promoting macrophage migration, we initially determined the signaling mechanism by which iFGFR1 regulates CX3CL1 gene and protein expression. Previous studies demonstrated that NFκB binds the proximal CX3CL1 promoter to drive expression [41], [45]. In accordance with these findings, we examined the ability of iFGFR1 to signal through NFκB and regulate CX3CL1. Treatment of HC-11/R1 cells with B/B to activate iFGFR1 elevated the transcriptional regulatory activity of NFκB. Furthermore, treatment of HC-11/R1 cells with B/B in the presence of the NFκB inhibitor peptide SN50 resulted in reduction of CX3CL1 gene expression. These findings depict a significant role for NFκB in mediating iFGFR1-regulated CX3CL1 expression. As a result, the NFκB pathway may provide a targetable approach for inhibition of iFGFR1-mediated CX3CL1 gene and protein expression in order to reduce macrophage migration and potential tumor growth and progression.

Understanding whether CX3CL1 contributes to early stages of mammary tumorigenesis is of great importance since CX3CL1 may serve as a potential biomarker for breast cancer risk and overall patient prognosis. CX3CL1 gene expression in normal breast epithelium has the potential to indicate patient susceptibility to developing breast cancer as well as risk of specific tumor molecular subtypes [46]. One study examined gene expression in normal breast epithelium found adjacent to malignant tissue in women with both estrogen receptor (ER) negative and positive breast cancer. Results demonstrated that CX3CL1 gene expression is induced in normal breast epithelium of ER− tumors compared to normal breast epithelium of ER+ tumors, suggesting that CX3CL1 expression may be an early marker of ER− tumor formation [46]. Furthermore, these data are supported by evidence from previously published breast cancer gene expression data sets in which increased CX3CL1 gene expression was linked to ER- tumors [47], [48], [49]. Conversely, recent studies demonstrated that while CX3CL1 expression did not significantly correlate with ER status, high levels of CX3CL1 was associated with better patient outcome [50]. Further studies are clearly required to fully understand whether CX3CL1 expression may help define patient risk and aid in distinguishing between susceptibility to molecular tumor subtypes during early cancer development before histological abnormalities are detected.

In addition to studying CX3CL1, previous studies have examined expression of CX3CR1 on breast cancer cells and the autocrine effects of the CX3CL1/CX3CR1 axis on regulating breast cancer cell migration. For instance, it was shown that exposure to proinflammatory cytokines increased the expression of CX3CR1 on human breast cancer cells thereby enhancing migration of these cells toward CX3CL1 [51]. Specifically, TNFα caused a significant increase in mRNA transcript levels of CX3CR1 in both MCF7 (ER+) and MDA-MB-231 (ER−) cells [51]. Moreover, treatment of MCF7 cells, which are minimally invasive and have low metastatic potential, with interleukin-1 and TNFα increased cell surface expression of CX3CR1 [51]. It has also been shown that CX3CR1 is involved in homing of breast cancer metastases to the brain [27]. Thus, expression of CX3CR1 in tumor cells may serve as a predictor for the occurrence of brain metastases [27]. Despite what is known about the autocrine effects of CX3CL1/CX3CR1, minimal research has been done to examine the role of CX3CL1 secretion by tumor cells [30] and the paracrine mechanisms by which tumor cell-secreted CX3CL1 interacts with CX3CR1-expressing cells of the surrounding tumor microenvironment during early mammary tumor formation.

In this paper we have shown for the first time that iFGFR1-induced CX3CL1 regulates the migration of macrophages during the initial stages tumor formation. Since tumor associated macrophage infiltration has previously been shown to correlate with poor patient prognosis in numerous tumor types, including breast tumors [16], it is important to identify the macrophage population that is present at the primary tumor site and what regulates the recruitment of this population. By blocking CX3CR1 *in vivo* we were able to reduce macrophage infiltration to the mammary epithelium of MMTV-iFGFR1 mice. Our previous studies using global macrophage depletion had demonstrated that loss of macrophages correlated with reduced epithelial cell proliferation and angiogenesis [15]. The results presented here demonstrate that blocking macrophage correlates with decreased angiogenesis, but not proliferation. These results suggest that global macrophage depletion, which was performed in the previous study and includes depletion of resident mammary gland macrophages, may have different effects on mammary tumorigenesis than the depletion of a population of infiltrating macrophages. It is also possible that the CX3CR1 blocking antibody is directly inhibiting blood vessel formation by blocking CX3CR1 expressed on endothelial cells [52], [53]. Further studies are required to determine the precise mechanisms through which blocking the CX3CL1/CX3CR1 axis regulates macrophage recruitment and angiogenesis in this system.

While our studies have focused specifically on macrophage recruitment, CX3CL1 is known to bind to a number of other immune cell types, including T cells, NK cells and dendritic cells [50]. Recent studies of breast cancer tissue samples demonstrated that CX3CL1 expression correlates with increased anti-tumor immune cells, including CD8+ T cells, NK cells and Cd1a+ dendritic cells, which correlated with better patient prognosis [50]. However, the link between FGFR activity, CX3CL1 expression and macrophage infiltration, and how these correlate with breast cancer subtype and patient outcome remain to be further determined. Because CX3CL1 can bind to a wide variety of cell types, including immune cells, endothelial cells and tumor cells [54], [55], elucidating the different mechanisms by which CX3CL1 acts on various cell types to regulate tumor formation and progression, either positively or negatively, is critical for fully understanding its likely complex role in the tumor microenvironment.

In conclusion, these study findings indicate a novel mechanism by which FGFR activation in mammary tumor cells promotes macrophage recruitment via induction of CX3CL1. Increased macrophage recruitment is associated with tumor growth and progression and is associated with poor prognosis of breast cancer patients [3], [16], [17], [18], [19]. Therefore, the identification of targetable factors that induce macrophage infiltration into the tumor microenvironment may lead to more effective novel therapeutic strategies that can be used in combination with tumor cell-targeted therapies. While further studies are required to fully understand the contributions of the CX3CL1/CX3CR1 axis to breast cancer, these results suggest that blocking CX3CL1/CX3CR1 interactions may provide a novel strategy for suppressing macrophage recruitment and the subsequent tumor promoting inflammation that occurs upon macrophage infiltration.

## Materials and Methods

### Ethics Statement

Animal care and experimental procedures were approved by the Institutional Animal Care and Use Committee of the University of Minnesota and were in accordance with the procedures detailed in the Guide for Care and Use of Laboratory Animals. The IACUC approval number is 0912A75315 and the date of approval was 02/21/12.

### Cell Culture

HC-11/R1 cells were generated and characterized previously [6] and were obtained as a gift from Dr. Jeff Rosen (Baylor College of Medicine, Houston, TX, USA). RAW 264.7 Cells and HS578T Cells were obtained from the American Type Culture Collection (ATCC, Manassas, VA, USA) and were maintained in DMEM (Invitrogen, Carlsbad, CA, USA) supplemented with 10% fetal bovine serum (FBS) (Invitrogen) and 1% penicillin-streptomycin (Invitrogen). Human THP1 cells were also previously obtained from the ATCC and were cultured in serum free (SF)-RPMI (Invitrogen) supplemented with 10% fetal calf serum (FCS), and 1% penicillin-streptomycin. THP1 cells were treated with phorbol myristate acetate (PMA) (P8139, Sigma-Aldrich, St. Louis, MO, USA) for 24 hours to differentiate them into macrophages as described previously [36], [56]. Cells were then washed with phosphate buffered saline (PBS) to remove trace amounts of PMA and were serum starved overnight. All cells were grown at 37°C under 5% CO_2_ atmosphere.

### Mass Spectrometry

HC-11/R1 cells were cultured into three 10 cm plates and grown to near confluence. They were washed three times with sterile PBS and incubated overnight in SF-RPMI media. Cells were treated with 30 nM B/B Homodimerizer (Clontech, Mountain View, CA, USA) in SF-RPMI. After 24 hours, approximately 30 mL of conditioned medium was collected in the presence of complete mini protease inhibitor cocktail with EDTA (Roche, Indianapolis, IN, USA) and stored on ice. The conditioned medium was concentrated to approximately 100 µL by centrifugation using 10 kDa molecular cutoff centrifugal filters (Millipore, St. Louis, MO, USA). After centrifugation, the protein component was precipitated with addition of 900 µL of ice-cold acetone and stored at −20^o^C overnight. The resulting pellet was washed three times with ice-cold acetone by repeated mixing by vortex and inversion followed by micro-centrifugation for 5 min at 10,000 rcf at 4^o^C. The pellet was dissolved in 0.1 M HEPES buffer pH 7.2 containing 4 M Urea. The resulting protein concentration was measured by BCA assay (Thermo Fisher Scientific, Pittsburg, PA, USA). Protein was reduced with addition of 2 mM TCEP (Thermo Fisher Scientific) for 30 minutes at room temperature and alkylated with 10 mM of Iodoacetamide (Sigma-Aldrich) for 45 minutes at room temperature in the dark. Protein was digested with addition of sequencing grade trypsin (Promega, Madison, WI, USA) at a 1∶50 enzyme:substrate ratio overnight at 37^o^C. Resulting peptides were purified using stage tips [57]. The acetonitrile from the eluate was removed by drying with vacuum centrifugation. The peptide samples were then dissolved in 98∶2:0.1 H_2_0:acetonitrile:formic acid and subjected to reversed phase nano-electrospray mass spectrometric analysis. Approximately one microgram was loaded and peptides were eluted over a 60-minute gradient of increasing acetonitrile from 4 to 40%. Mass spectrometry and database searching were performed as previously described [58]. A composite database of the canonical mouse protein database from www.uniprot.org, from 08/19/10, its reversed counterpart, and a list of common contaminants were used for peptide identification using semi-tryptic specificity, methionine oxidation as a variable modification, carbamidomethyl cysteine as a fixed modification. Protein identifications were filtered down to less than 1% false discovery rate using a 10 ppm precursor mass tolerance window and at least two peptides identified per protein. Peptide probabilities [59] were calculated using Scaffold 3 (www.proteomesoftware.com). The analyses were done three different times using three different cell preparations.

### ELISA Analysis

HC-11/R1 cells were grown to confluence and incubated overnight in SF-RPMI media. Following serum starvation, cells were treated with 30 nM B/B Homodimerizer (Clontech, Mountain View, CA, USA) or an equal amount of ethanol as a solvent control. Conditioned medium was harvested from cells after 24 hours, unless otherwise noted. Conditioned media samples were analyzed using an ELISA (MCX310, R&D Systems, Minneapolis, MN, USA) to quantify the concentration of CX3CL1 secreted by HC-11/R1 cells in the presence of iFGFR1 activation.

### RNA Isolation and Quantitative RT-PCR Analysis

Trizol isolation was used to harvest RNA from monolayer cells in culture in accordance with procedures recommended by the manufacturer (Invitrogen). cDNA was synthesized from RNA using the QuantiTect Reverse Transcription kit (Qiagen, Valencia, CA, USA). One-tenth of the final cDNA reaction volume was then used in quantitative SYBR (Synergy Brands) green RT-PCR reactions as described previously [60]. SYBR green RT-PCR reactions were performed using the Bio-Rad iQ5 system (Bio-Rad, Hercules, CA, USA). Expression of each gene was calculated and normalized to average cyclophilin expression levels as indicated using the 2^−ΔΔCt^ method [60]. For analysis of murine CX3CL1 mRNA levels, HC-11/R1 cells were serum-starved overnight and then treated with 30 nM B/B Homodimerizer (Clontech) or ethanol solvent control. For analysis of human CX3CL1 mRNA levels, HS578T cells were serum-starved overnight and then treated with 50 ng/mL basic FGF (bFGF) (Invitrogen) or PBS solvent control for 8 hours. The following primer sequences were used: murine CX3CL1 5′-CTGGCCGCGTTCTTCCATT-3′ and 5′-GATTTCGCATTTCGTCATGCC-3′, murine cyclophilin 5′-TGCAGGCAAAGACACCAATG-3′ and 5′-GTGCTCTCCACCTCCCGTA-3′, human CX3CL1 5′- ACCACGGTGTGACGAAATG -3′ and 5′-TGGATGAGCAAAGCTACAGGTA-3′, and human cyclophilin 5′-GAAAGAGCATCTACGGTGAGC-3′ and 5′-GTCTTGACTGTCGTGATGAAGAA-3′. All statistical analyses were performed using the unpaired student’s t-test to compare two means.

### NFκB Luciferase Assay

A Cignal NFκB reporter assay kit (336841, Qiagen, Valencia, CA, USA) was used to monitor the activity of NFκB-regulated signal transduction in HC-11/R1 cells. This reporter contains a mixture of an inducible NFκB-responsive luciferase construct and a constitutively active Renilla construct. Therefore, a Dual-Luciferase Reporter Assay (E1910, Promega) was performed to efficiently measure NFκB-responsive luciferase activity in accordance with the protocols recommended by the manufacturer.

### NFκB Inhibitor

HC-11/R1 cells were serum starved overnight and then treated with 30 nM B/B, or ethanol as the solvent control, in the presence of 50 ug/mL NFκB SN50, Cell-Permeable Inhibitor Peptide (481480, EMDbiosciences, San Diego, CA, USA) or 50 ug/mL NFκB SN50M, Cell-Permeable Inactive Control Peptide (481486, EMDbiosciences) for 4 hours.

### Animals

Generation of mouse mammary tumor virus (MMTV)-iFGFR1 transgenic mice has been described previously [38]. MMTV-iFGFR1 mice were obtained from Dr. Jeff Rosen (Baylor College of Medicine, Houston, TX, USA).

### Treatment of Mice

Six-week old female mice were injected intraperitoneally (i.p.) with 75 uL of purified polyclonal rabbit anti-CX3CR1 antibody (TP501, Torrey Pines Biolabs, San Diego, CA, USA) or with control rabbit immunoglobulin (Ig) G (026102, Invitrogen) at a concentration of 20 ug/mL for 24 hours prior to treatment with 1 mg/kg B/B and in conjunction with administration of B/B for 10 days. Non-transgenic littermate control mice were also included in both the IgG control group and the anti-CX3CR1 experimental study group. Two hours before sacrifice, the mice were given i.p. injections of 0.3 mg/kg BrdU (GE Healthcare Life Sciences, Piscataway, NJ, USA). Mice were sacrificed after 10 days of B/B treatment and mammary glands from at least three mice were analyzed for each treatment group.

### Microarray Analysis

Six-week-old female MMTV-iFGFR1 transgenic mice and non-transgenic littermates were injected intraperitoneally (i.p.) with 1 mg/kg B/B (Clontech). Mice were sacrificed after 48 hours or 4 weeks of B/B treatment and mammary glands were collected for analysis. The tissue was dissociated using 2 mg/ml collagenase A (Roche Applied Science, Indianapolis, IN, USA) for 45 minutes at 37°C with rocking at 200 rpm. The solutions were vigorously shaken every 15 minutes and the dissociated cells were collected by centrifuging for 5 minutes at 1500 rpm. The cells were washed 3 times with DMEM/F12 containing 5% FCS at 1500 rpm and 2 times at 800 rpm for 5 minutes each. The cells were stained with either Cd11b-APC (Life Technologies, Grand Island, NY, USA) at a dilution of 1∶200 or isotype control antibody at the same concentration for 1 hour at RT. The cells were then washed, filtered through a 40 micron filter and sorted using a triple laser MoFlo (Cytomation, Fort Collins, CO). RNA was isolated from Cd11b-positive cells sorted from 6 mice per timepoint and pooled into duplicate samples. RNA was extracted using the Arcturus PicoPure RNA Isolation Kit (Life Technologies) and hybridized to the Affymetrix MOE 2.0 microarray (Affymetrix, Santa Clara, CA, USA) in the Baylor Microarray Core Facility at Baylor College of Medicine (Houston, TX). Raw data were normalized using Microarray Suite 5.0 and the normalized data were analyzed both Genespring (Silicon Genetics, Palo Alto, CA). Genes called absent in all samples were discarded and genes that were either upregulated or downregulated at least 2-fold with a p-value of less than 0.05 were further analyzed using Integrated Pathways Analysis (IPA) (Ingenuity Systems, Redwood City, CA, USA). Data have been deposited in the National Center for Biotechnology Information Gene Expression Omnibus data repository, accession number GSE3647 (Reviewer link: http://www.ncbi.nlm.nih.gov/geo/query/acc.cgi?token=xbwxfoyiymuwgvi&acc=GSE36477).

### Immunofluorescence

Mammary glands were fixed for two hours in 4% paraformaldehyde and embedded in paraffin. For immunofluorescent analysis, mammary glands were sectioned and stained with F4/80 (MF48000, Invitrogen) at a 1∶50 dilution. No antigen retrieval was performed for immunohistochemical analysis of tissue sections with F4/80. Proliferation was quantified with anti-BrdU (ab6326, Abcam) at a 1∶300 dilution in TNBTT. F4/80 and BrdU Antibody staining was visualized with Alexa Fluor 594 Goat anti-Rat IgG (A11007, Invitrogen) at a 1∶250 dilution, and sections were counter stained with DAPI (P36931, Invitrogen). F4/80 and BrdU positive cells were counted and calculated relative to the number of total epithelial cells. Blood vessel formation was quantified using anti-vWF (A0082, DAKO) at a 1∶400 dilution. vWF antibody staining was visualized with Alexa Fluor 488 Goat anti-Rabbit IgG (A11008, Invitrogen) at a 1∶200 dilution, and sections were counter stained with DAPI. Blood vessels that were less than 20µM and associated with epithelial ducts were counted and calculated relative to the number of total epithelial cells [15]. A minimum of 1000 epithelial cells from three mice per treatment group was assessed for each stain. All statistical analyses were performed using the unpaired student’s t-test to compare two means.

### CX3CL1 siRNA

Reduction of CX3CL1 in HC-11/R1 cells was performed via the mechanism of RNA interference in accordance with procedures recommended by the manufacturer (Thermo Fisher Scientific). In brief, HC-11/R1 cells were transfected with 50 nM final concentration of ON-TARGET *plus* non-targeting pool (D-001810-10, Thermo Fisher Scientific) or 50 nM final concentration of ON-TARGET *plus* SMARTpool mouse CX3CL1 siRNA (L-0623510-01, Thermo Fisher Scientific). DharmaFECT (T-2001-02, Thermo Fisher Scientific) transfection reagent was used to deliver siRNA into cultured HC-11/R1 cells.

### Transwell Chemotaxis Assays

8-µm sized pore inserts (Falcon; BD Biosciences) were placed in 24-well plates. Serum starved RAW 264.7 cells were loaded into the insert at a density of 20,000 cells per 0.5 mL of serum free media. Each chamber of the 24-well plate contained 0.75 mL of conditioned medium from either HC-11/R1 cells treated with B/B or ethanol solvent control or conditioned medium HC-11/R1 cells transfected with either non-targeting siRNA control or CX3CL1 siRNA and then treated with ethanol solvent control, B/B, or 50 ng/mL recombinant mouse CX3CL1 (rmCX3CL1) and B/B for 24 hours. Serum starved, differentiated THP1 cells were loaded into the insert at a density of 20,000 cells per 0.5 mL of serum free media and were either untreated or treated with 0.5 ug/mL of goat antiCX3CL1 (AF365, R&D Systems) or goat IgG control (AB-108-C, R&D Systems). Each chamber of the 24-well plate contained 0.75 mL of conditioned media from confluent HS578T cells grown in complete media and treated with PD173074 or DMSO solvent control as well as conditioned media from confluent HS578T cells grown in complete media or serum free control. Cells were then incubated at 37°C for 24 hours. After 24 hours, cells that migrated through the pores of the insert were fixed in 4% paraformaldehyde (PFA) and stained with hematoxylin. Migration was determined based on the number of cells that traveled through the pore inserts and stained positively with hematoxylin.
